# Rare Causes of Acute Coronary Syndrome: Carbon Monoxide Poisoning

**DOI:** 10.3390/life12081158

**Published:** 2022-07-29

**Authors:** Raluca Ecaterina Haliga, Bianca Codrina Morărașu, Victorița Șorodoc, Cătălina Lionte, Oana Sîrbu, Alexandra Stoica, Alexandr Ceasovschih, Mihai Constantin, Laurentiu Șorodoc

**Affiliations:** 1 Department of Internal Medicine and Toxicology, Saint Spiridon University Regional Emergency Hospital, 700111 Iasi, Romania; raluca.haliga@umfiasi.ro (R.E.H.); victorita.sorodoc@umfiasi.ro (V.Ș.); catalina.lionte@umfiasi.ro (C.L.); oana.sirbu@umfiasi.ro (O.S.); alexandra.rotariu@umfiasi.ro (A.S.); alexandr.ceasovschih@umfiasi.ro (A.C.); mihai.s.constantin@umfiasi.ro (M.C.); laurentiu.sorodoc@umfiasi.ro (L.Ș.); 2 Faculty of Medicine, “Grigore T. Popa” University of Medicine and Pharmacy, 700111 Iasi, Romania

**Keywords:** acute coronary syndrome, carbon monoxide poisoning, myocardial injury, severity of poisoning

## Abstract

Acute coronary syndrome (ACS) is a spectrum of clinical and paraclinical disorders arising from an imbalance of oxygen demand and supply to the myocardium. The most common cause is atherosclerosis; however, other rare causes such as carbon monoxide (CO) poisoning should be considered. Through tissue hypoxia and direct cell injury, CO poisoning can lead to a broad spectrum of cardiac disorders, especially ACS. **Materials and Methods**. We have conducted a retrospective study in the Toxicology Department of Saint Spiridon Emergency University Hospital, including all patients admitted through the emergency department with CO poisoning. We divided the cohort into event group (myocardial injury) and non-event group (patients without myocardial injury) and performed a subset analysis of the former. **Results**. A total of 65 patients were included, 22 in the event and 43 in the non-event group. The severity of poisoning did not correlate with myocardial injury; however, 50% of the event group had severe poisoning with carboxyhaemoglobin ≥ 20%. Cardiac enzyme markers (troponin and creatin-kinase MB) had a statistically significant increase in the event group compared to the non-event group (*p* < 0.05). Most of the patients in the STEMI (50%) and NSTEMI (66.7%) groups had severe CO intoxication. The STEMI group had a mean age of 27.7 years old and no comorbidities. **Conclusions**. Myocardial injury can develop in CO poisoning irrespective of the severity of poisoning, and it can be transient, reversible, or permanent. Our study introduces new information on adverse cardiac events in patients with CO poisoning, focusing on the ACS. We found that the severity of CO poisoning plays an important role in developing myocardial injury, as 50% of patients in the event group were severely intoxicated. While in-hospital mortality in our study was low, further prospective studies should investigate the long-term mortality in these patients.

## 1. Introduction

Acute coronary syndrome (ACS) is a consequence of a sudden imbalance between oxygen demand and supply to the myocardium. It is represented by a spectrum of clinical and paraclinical presentations, ranging from ST-segment elevation myocardial infarction (STEMI) to non–ST-segment elevation myocardial infarction (NSTEMI) or unstable angina [1]. The most common cause of ACS is atherosclerosis (90%), which is frequently complicated, leading to partial or complete thrombosis of infarct-related coronary artery [2]. There are other rare causes of ACS, such as endocrine or haematological. Spasm, obstruction, inflammation, or trauma of the coronary arteries can cause myocardial injury. Carbon monoxide (CO) poisoning, through different mechanisms, can be the cause of ACS.

CO poisoning is a major public health problem, being one of the leading causes of death and injury worldwide [3]. Cumulative worldwide incidence and mortality of CO poisoning are currently estimated at 137 cases and 4.6 deaths per million, respectively [4]. In Europe, national data provided by 28 European member states on CO poisoning reported an annual rate of 2.2/100,000 CO-related deaths [5].

CO has a specific effect on highly sensitive tissues to hypoxia, such as brain or heart [6]. Its toxicity is the result of hypoxia, increased carboxyhaemoglobin (CoHgb) formation and direct CO-mediated cell damage. Furthermore, CO can induce coronary spasm and intracoronary thrombosis and increase vascular permeability and platelet aggregation [7], which can lead to ACS both on healthy and non-critical atherosclerotic plaque [8].

In this retrospective study, we aim to analyse and describe our experience regarding cardiovascular events in the context of acute CO poisoning, focusing on ACS in relation to the severity of CO intoxication, comorbidities, clinical and biological characteristics, and outcome of patients.

## 2. Materials and Methods

### 2.1. Study Design and Selection of Patients

We carried out a retrospective study in “Saint Spiridon” Regional Emergency Hospital, Toxicology Department, a tertiary regional centre for clinical toxicology in northeast Romania. We included all patients aged over 18 with a diagnosis of CO poisoning between 1 January 2013 and 31 December 2021.

Inclusion criteria consisted of patients admitted through the emergency department (ED) with at least one of the following: history of CO exposure, increased COHgb levels, and symptoms consistent with CO poisoning.

The electronic hospital records were searched based on the patient’s diagnosis at discharge. We extracted the following data: patient’s demographics (gender, age, residential area, smoking status), body mass index (BMI—using the following cut-off: normal—18.5–24.9 kg/m^2^, overweight—25–29.9 kg/m^2^, obesity ≥ 30 kg/m^2^) [9], comorbidities (arterial hypertension, ischaemic heart disease, previous myocardial infarction (MI), heart failure, diabetes mellitus type 2, presence of angina type pain, level of COHgb on admission, cardiac enzymes (troponin and creatin-kinase MB (CK-MB)), electrocardiogram (ECG) upon presentation and during admission, if performed by attending physician, need for orotracheal intubation upon presentation, clinical outcome and length of admission. ECGs were retrospectively reviewed by 2 Internal Medicine Trainees (1st and 5th year of training). Subsequently, an Internal Medicine Consultant randomly reviewed the ECGs to ensure accuracy.

We used the following definitions for the severity of poisoning: mild poisoning—COHgb > 10% without clinical signs and symptoms or COHgb < 10% with associated signs and symptoms, moderate poisoning—COHgb 10–19% with clinical signs and symptoms, severe poisoning—COHgb ≥ 20% with clinical signs and symptoms [3,10]; for clinical outcome: favourable—the patient was discharged when considered clinically improved by treating physician; against medical advice—the patient discharged himself against medical advice from treating physician; death—patient died due to CO poisoning or associated acute illness in this context. Length of admission was divided into 3 main categories: 1–3 days; 4–7 days and >7 days. Whenever the information was missing, it was mentioned as not available (NA).

Patients younger than 18, with acute concomitant illness requiring a specific approach in a different department, except for the Toxicology Intensive Care Unit (ICU) (e.g., burns, trauma, acute stroke etc.), were excluded from our study.

### 2.2. Outcomes

The primary outcome was to analyse the incidence and types of cardiac ischemic events by dividing the patients into 2 cohorts: event group (myocardial injury) and non-event group (patients without myocardial injury). We further performed a subset analysis of patients in the event group in relation to the severity of acute CO poisoning. Myocardial injury was identified as evidence of STEMI (new ST-segment elevation ≥ 1 mm in 2 consecutive leads), NSTEMI (new ST-segment depression ≥ 0.5 mm in 2 consecutive leads), other features of ischaemia (new T-wave inversion ≥ 2 mm in 2 consecutive leads). Secondary outcomes were the presence of rhythm and conduction disorders, positive cardiac enzymes, need for intubation, length of admission and clinical outcome.

### 2.3. Blood Analysis

COHgb concentration in venous blood was measured using the ABL90 Series method (Radiometer, Copenhagen, Denmark) in ED. Cardiac enzymes (troponin and CK-MB) were measured using an ARHITECT clinical chemistry analyser (Abbott Laboratories, Abbott Park, IL, USA). This high-sensitivity troponin assay was introduced in 2018. Throughout the years, we have used several assays with different sensitivities and specificities.

### 2.4. Treatment

All patients underwent initial assessment and treatment in the ED to ensure the stability of vital signs, carboxyhaemoglobin measurement and 100% oxygen therapy, as well as full blood tests (full blood count, biochemistry, coagulation tests) in symptomatic patients (e.g., malaise, nausea, headache, chest pain, reduced consciousness, coma [3]). Cardiac enzymes were taken as indicated by the emergency medicine/internal medicine/toxicology consultant. In the case of severe poisoning associated with the need for intubation (GCS 8, acute respiratory failure not corrected by non-invasive treatment), severe acidosis (pH < 7.2 despite adequate treatment), fluid-resistant hypotension (MAP < 65 mmHg), or other appropriate criteria, as indicated by the ICU Consultant, patients were managed in the Toxicology ICU department. Those clinically stable (e.g., systolic blood pressure > 90 mmHg, GCS > 9, oxygen saturation > 80 correcting with oxygen therapy, absence of new organ failure) but requiring further observation and/or management of CO poisoning and associated comorbidities were admitted to our Toxicology Department.

### 2.5. Data Analysis

Statistical analyses were performed with SPSS software for Windows (v.22.0; SPSS, Chicago, IL, USA). Nominal variables are presented as frequencies and percentages, and continuous variables are presented as the mean ± standard deviation (SD). To identify significant parameters associated with a CO poisoning diagnosis, a two-tailed Student’s *t*-test was used to compare normally distributed continuous variables, whereas the chi-squared test and Cochrane’s statistic were used for categorical variables. A *p*-value < 0.05 was considered statistically significant.

## 3. Results

### 3.1. Patients’ Characteristics

Sixty-five patients with acute CO poisoning were admitted to our Toxicology department between 2013 and 2021, out of a total of 1780 patients with drug/non-pharmaceutical-induced acute poisonings. In all cases, the poisoning was accidental because of either a house fire, incorrectly installed, or faulty gas-powered generators, heating systems or wood stoves. The demographic characteristics of the cohort are presented in Table 1.

A high percentage (40%) of patients were young, aged between 18 and 39 years old. In total, 55.4% of patients were female, 61.5% lived in urban areas, and 35.4% were current smokers.

### 3.2. Severity of CO Poisoning

Of the 65 acute CO poisoning patients included in the study, 44.6% had mild, while 35.4% had severe poisoning.

### 3.3. Cardiac Ischemic Events

Out of 65 patients, 22 are in the event group and 43 in the non-event group, with a mean age of 57 years old and 48 years old, respectively (Table 2).

Regarding smoking status and comorbidities, there is no statistically significant difference between the two groups. Approximately one-third of the patients were current smokers in both groups. Comorbidities were present in both groups to a certain degree; arterial hypertension was slightly more present in the event group (27.3%) compared to the non-event group (14.0%).

The severity of poisoning does not correlate with cardiac ischaemic events. For each degree of CO toxicity, there is a similar proportion of affected individuals, both in the event and non-event groups. However, 50% of the event group had severe poisoning with COHgb ≥ 20%, while the non-event group had mild poisoning in most cases (48.8%).

Cardiac enzyme markers (troponin and CK-MB) had a statistically significant increase in the event group compared to the non-event group (*p* < 0.05). On the other hand, troponin was not available in 58.1% of the non-event group. There was no difference between groups regarding orotracheal intubation.

Patients with cardiac events had a longer admission, 4–7 days (45.5%), compared to the non-event group, admitted for 1–3 days (55.8%). Most of our patients had a favourable outcome, 77.3% in the first and 55.8% in the second group, with 9.1% and 7% mortality rates in the two groups.

We further characterised the event group depending on the type of ECG changes (other features of ischaemia, STEMI and NSTEMI). There are nine patients with other features of ischaemia, four patients with STEMI and nine with NSTEMI. The STEMI group is relatively younger, with a mean age of 27.7 years old and no comorbidities. Arterial hypertension and ischaemic heart disease are present in 44.4% and 33.3% of the other features of the ischaemia group, compared to 22.2% and 11.1% in the NSTEMI group. Most patients in other features of the ischaemia group (44.4%) had mild poisoning, while 66.7% of the NSTEMI (66.7%) group had severe CO poisoning (Table 3).

Troponin was increased in the other features of ischaemia and NSTEMI groups, but the majority of patients had normal levels. In total, 50% of STEMI and 33% of the NSTEMI population had an increase in CK-MB levels. Half of the patients with STEMI were intubated. The length of admission was equally distributed in the NSTEMI group; 4–7 days for most (55.6%) of the other features of the ischaemia group, and 1–3 days for STEMI. The highest mortality was 25% (*n* = 1) in patients with STEMI and lower (11%) in the other two groups (Table 3).

### 3.4. Arrhythmias

They were described as more frequent (17.2%) in mild CO poisoning, being represented by the new appearance of either sinus tachycardia, atrial or ventricular extrasystoles, or atrial fibrillation (Table 4).

Furthermore, ECG atrioventricular (AV) conduction disorders (newly diagnosed bundle branch blocks) were more frequently described (17.2%) in mild CO poisoning. Both arrhythmias and conduction disorders did not show statistical significance depending on CO poisoning severity (Table 4).

## 4. Discussion

Our study comprises a relatively young cohort with no significant comorbidities and mild poisoning. The severity of poisoning does not have a statistically significant correlation with myocardial injury. On the other hand, one-third of the studied population had either STEMI, NSTEMI or other features of ischaemia on the ECG, and half of them had severe poisoning. Most patients had normal troponin levels. Those with mild poisoning developed arrhythmias (17.2%). This highlights the heterogeneity of CO poisoning patients. They may develop myocardial injury in the absence of significant risk factors. Hence, an individualised approach is necessary when treating CO poisoning. Moreover, future research should address the long-term complications and establish the need for follow-up in this special category of patients.

### 4.1. Cardiovascular Effects of CO Poisoning

There is a high variability of myocardial involvement based on the cardiovascular status of the affected individual. In most cases, a severe degree of CO poisoning can cause acute coronary syndrome, even in individuals with minimal or no coronary atherosclerosis. However, there are cases in the literature reporting that mild CO poisoning can also lead to severe myocardial injury, especially in older patients with cardiovascular risk factors [11]. Patients may develop angina, which can trigger the physician to further investigate as this symptom seems to correlate with the degree of CO poisoning [12]. On the other hand, cardiac chest pain can develop later in the course of the disease [13], or it can be completely absent [14]. Overall, 13% of our event group developed angina compared to 2.3% in the non-event group. Another study showed that of 104 patients referred to the coronary care unit with unstable angina, three patients had chronic CO poisoning, and five had exposure. This suggests that even environmental CO exposure can result in high COHgb concentrations, causing acute coronary syndrome. In most cases, patients are investigated in the context of acute intoxication; otherwise, this may be overlooked by the physician [15].

There were no major differences in comorbidity profiles between the event and non-event groups. Out of arterial hypertension, diabetes mellitus, ischaemic heart disease, previous MI and heart failure, only the former was slightly more frequent in the event group. These patients do not have the common risk factors of patients with cardiovascular disease, as demonstrated by another retrospective study with a 230 CO poisoning cohort where cardiovascular comorbidities were uncommon, with only 7% of patients having previous MI. Interestingly, arterial hypertension was identified as a predictor of myocardial injury [16].

Smoking is a well-known cardiovascular risk factor determining around 30% of coronary artery disease mortality. [17]. Around 40% of our cohort were active smokers, with no significant difference between the event and non-event group. We can argue that different types of smoking expose individuals to CO, and their baseline COHgb may increase to 10%. This chronic exposure can cause symptoms of fatigue or headache that may worsen in the context of acute exposure and accelerate ischaemic events [18].

It is difficult to determine whether a patient will develop myocardial injury and most studies do not report a statistically significant correlation between levels of COHgb and adverse cardiovascular events. Our study is consistent with these previous findings as the severity of poisoning did not correlate with cardiac ischaemic events. On the other hand, ECG changes and insufficient diagnostic performance of cardiac enzymes may be misleading in appreciating the degree of cardiac impairment. Ischaemic changes have been described in several case reports until larger cohorts were studied. Anderson [19] reports ST segment abnormalities (elevation and depression) in a case series with seven patients but apical thrombus and coronary artery thrombosis in one patient. From a total of 250 patients, Cha YS et al. [20] described ischaemic changes in 3.6% of patients (ST elevation in 0.8%, ST depression 1.6% and T wave inversion in 1.2%), a relatively low incidence, but included patients with less severe CO poisoning (mean initial COHgb was 13.55%). We determined that out of all patients in the event group, 50% had severe poisoning with a COHgb ≥ 20%. In the subset analysis of our event group, nine patients had other features of ischaemia, nine had NSTEMI and four of them STEMI. The ECG changes were more frequent in those with NSTEMI and severe poisoning (66.7%). Most patients in the other features of the ischaemia group (44.4%) and STEMI group (50%) had mild poisoning. Another study showed that 85 out of 230 patients developed ST and T wave changes consistent with myocardial injury, and 35% had increased cardiac enzymes [16]. Our STEMI group had no increase in troponin levels. Although troponin, especially high sensitivity troponin, is a key marker of myocardial injury, it cannot be used to predict cardiovascular events in the context of moderate to severe CO poisoning [21]. These changes may be transient and determined by direct CO toxicity, as opposed to atherosclerotic mechanisms. This hypothesis is confirmed by a prospective study that evaluated the cardiac function and structure of patients with CO poisoning. Those with elevated cardiac enzymes were explored with coronary angiography, which was normal in all cases. Coronary spasm was excluded by provocation tests. We can, therefore, argue that increased levels of CK-MB and troponin can arise in anatomically intact coronary arteries [22]. On the other side, STEMI due to left anterior descending coronary artery thrombosis [23], total occlusion of a branch of the right coronary artery [24] or myocardial rupture and tamponade secondary to ACS have also been described in CO poisoning [25].

In the subset analysis groups, most of the patients had normal troponin and CK-MB levels. Troponin was slightly increased in the other features of ischaemia and NSTEMI subgroups of the event cohort, and 50% of STEMI and 33% of the NSTEMI population had an increase in CK-MB levels. Cho et al. [26] found increased troponin in 54% of the 359 patients included in the study. A total of 104 patients underwent cardiac MRI, out of which 72 had late gadolinium enhancement with mid-wall myocardial injury. This cohort included patients with no cardiovascular comorbidities, similar to our STEMI group as our patients were relatively young, with a mean age of 27.7 years old, active smokers (50%), and no comorbidities. In addition to cardiac MRI, SPECT scintigraphy has been proved to provide a significant correlation between the imagistic changes and severity of CO poisoning [27].

Regarding the length of admission, patients in the event group had longer admission compared to the non-event group. Most of our patients had a favourable outcome on discharge. On the other hand, when followed up for a longer period of time, patients with myocardial injury due to CO poisoning may have a three times higher mortality compared to those without myocardial injury and CO poisoning [28]. Henry et al. [29] report that out of the patients with increased cardiac markers and ECG changes consistent with myocardial injury, 38% died after a 7.6-year follow-up. Half of our patients with STEMI were intubated. Length of admission was equally distributed in the NSTEMI group, with 4–7 days and for 55.6% of the ECG ischaemic changes group and 1–3 days for STEMI. The highest mortality was 25% (*n* = 1) in patients with STEMI and lower (11%) in the other two groups. Further studies need to investigate the long-term outcome of patients with cardiac events and CO poisoning.

### 4.2. Arrhythmias, Conduction Disorders

In our study, arrhythmias were represented by newly diagnosed sinus tachycardia, atrial and ventricular extrasystoles, and atrial fibrillation on ECG. These were more frequent in mild forms of acute CO poisoning. ECG intra-ventricular conduction disorders (newly diagnosed bundle branch blocks) were present in 25% of cases in mild forms, and 17.4% were present in severe forms of acute CO intoxication. Sinus tachycardia was reported in 9 out of 40 patients in one investigation by Aslan et al. [30], while in another study by the same author, it was noted in 26.5% of patients (*n* = 83) [31].

Lee et al. [32] showed in a cohort with 8381 CO poisoning patients that the risk of arrhythmia was twice as high in CO poisoning patients compared with those without CO poisoning. The risk remained significantly higher in patients with associated comorbidities and severe CO poisoning after the adjustment of confounders such as sex, age, or comorbidity. Systemic hypoxia in CO poisoning induces compensatory tachyarrhythmia, increases the oxygen demand, and accelerates CO diffusion, which further exacerbates hypoxic injury of the myocardium. A few cases of intra-ventricular conduction disorders associated with CO poisoning were reported. Most of them were reversible and explained by cardiac ischaemia and toxic effects of CO, such as the case of a young female presenting with a transient left bundle branch block and normal coronary angiography in the context of moderate CO poisoning [33].

### 4.3. Study Limitations

Our study has several limitations. It is a retrospective study with a review of medical records with some information being unavailable or missing. We conducted the study in a single centre, and the cohort had a relatively small number of patients. Cardiac markers were not available in all patients. Moreover, the troponin assay has changed throughout time, currently providing more accurate and earlier detection of cardiac injury. Since 2018, we have introduced high-sensitivity troponin. This might have affected the number of patients diagnosed with cardiac events prior to 2018, hence the number of patients included in our study.

## 5. Conclusions

Our study introduces new information on cardiac adverse events in patients with CO poisoning, focusing on the ACS. We found that the severity of CO poisoning plays an important role in developing cardiac ischaemic events, as 50% of patients in the event group were severely intoxicated. On the other side, none of the studied parameters correlated the degree of CO poisoning with the risk of developing myocardial injury.

Taking into account the variability found in our study, we can acknowledge that cardiovascular events in CO poisoning are difficult to predict. This is due to myocardial injury, which can be transient, reversible, or permanent and can arise in both intact or atherosclerotic coronary arteries. Routine investigations, such as ECG, cardiac enzymes or echocardiography, may not be sufficient. On the other hand, invasive and expensive investigations (coronary angiography, cardiac MRI) should be carefully sought as patients may exhibit transient changes. Our in-hospital mortality was low, but future prospective studies are needed to investigate the long-term effects of cardiac involvement at the time of poisoning.

## Figures and Tables

**Table 1:** Characteristics of patients with acute CO poisoning.

Demographic Data	Total (*n* = 65)	%
Age interval		
18–39 years	26	40.0
40–59 years	12	18.5
60–79 years	19	29.2
over 80 years	8	12.3
Gender		
Male	29	44.6
Female	36	55.4
Residence		
Urban	40	61.5
Rural	25	38.5
Smoking		
Current smoker	23	35.4
Non-smoker	42	64.6
Body mass index		
Normal	21	32.3
Overweight	7	10.8
Obesity	4	6.2
Not available	33	50.8
**Patients’ comorbidities**		
Diabetes mellitus type 2	7	10.8
Arterial hypertension	12	18.5
Ischemic heart disease	9	13.8
Previous MI	1	1.5
Heart failure	9	13.8
**Severity of poisoning**		
Mild poisoning	29	44.6
Moderate poisoning	13	20.0
Severe poisoning	23	35.4

**Table 2:** Characteristics of event and non-event groups.

Parameter	Groups		χ^2^ Test	T Test	*p* Value
	Event (*n* = 22)	Non-Event (*n* = 43)			
**Mean Age**	57.45 ± 24.27	48.65 ± 21.06		2.292	0.135
**Smoking status**			0.440		0.507
Current smoker	9 (40.9%)	14 (32.6%)			
Non-smoker	13 (59.1%)	29 (67.4%)			
**Angina**	3 (13.6%)	1 (2.3%)	3.029		0.082
**Comorbidities**					
Diabetes mellitus type 2	2 (9.1%)	5 (11.6%)	0.100		0.752
Arterial hypertension	6 (27.3%)	6 (14.0%)	1.646		0.200
Ischemic heart disease	4 (18.2%)	5 (11.6%)	0.507		0.477
Previous myocardial infarction	0 (0.0%)	1 (2.3%)	0.834		0.361
Heart failure	4 (18.2%)	5 (11.6%)	0.507		0.477
**Severity of poisoning**			3.153		0.207
Mild poisoning	8 (36.4%)	21 (48.8%)			
Moderate poisoning	3 (13.6%)	10 (23.3%)			
Severe poisoning	11 (50.0%)	12 (27.9%)			
**Troponin**			10.396		0.006
Increased	2 (9.1%)	1 (2.3%)			
Normal	16 (72.7%)	17 (39.5%)			
NA	4 (18.2%)	25 (58.1%)			
**CK-MB**			9.009		0.011
Increased	5 (22.7%)	13 (30.2%)			
Normal	16 (72.7%)	17 (39.5%)			
NA	1 (4.5%)	13 (30.2%)			
**Orotracheal** **intubation**	3 (13.6%)	5 (11.6%)	0.054		0.817
**Length of admission**			4.067		0.131
1–3 days	7 (31.8%)	24 (55.8%)			
4–7 days	10 (45.5%)	15 (34.9%)			
Over 7 days	5 (22.7%)	4 (9.3%)			
**Outcome**			4.260		0.119
Favourable	17 (77.3%)	24 (55.8%)			
Death	2 (9.1%)	3 (7.0%)			
Discharged against medical advice	3 (13.6%)	16 (37.2%)			

**Table 3:** Characteristics of the event group depending on the type of ECG changes.

Parameter	Other Features of Ischaemia (*n* = 9)	STEMI (*n* = 4)	NSTEMI (*n* = 9)	*p* Value for T Test	*p* Value for Cfi Square Test
**Mean Age**	58.56 ± 24.42	27.75 ± 9.74	69.56 ± 17.53	0.009	
**Smoking status**					0.308
Current smoker	3 (33.3%)	2 (50.0%)	3 (33.3%)		
Non-smoker	6 (66.7%)	2 (50.0%)	6 (66.7%)		
**Comorbidities**					
Diabetes mellitus type 2	1 (11.1%)	0 (0.0%)	1 (11.1%)		0.655
Arterial hypertension	4 (44.4%)	0 (0.0%)	2 (22.2%)		0.144
Ischemic heart disease	3 (33.3%)	0 (0.0%)	1 (11.1%)		0.210
Previous myocardial infarction	0 (0.0%)	0 (0.0%)	0 (0.0%)		-
Heart failure	2 (22.2%)	0 (0.0%)	2 (22.2%)		0.408
Severity of poisoning					0.482
Mild poisoning	4 (44.4%)	2 (50.0%)	2 (22.2%)		
Moderate poisoning	2 (22.2%)	0 (0%)	1 (11.1%)		
Severe poisoning	3 (33.3%)	2 (50.0%)	6 (66.7%)		
**Troponin**					0.855
Increased	1 (11.1%)	0 (0.0%)	1 (11.1%)		
Normal	7 (77.8%)	2 (50.0%)	6 (66.7%)		
NA	1 (11.1%)	2 (50.0%)	2 (22.2%)		
**CK-MB**					0.095
Increased	0 (0.0%)	2 (50.0%)	3 (33.3%)		
Normal	8 (88.9%)	2 (50.0%)	6 (66.7%)		
NA	1 (11.1%)	0 (0.0%)	0 (0.0%)		
**Orotracheal** **intubation**	1 (11.1%)	2 (50.0%)	1 (11.1%)		0.791
**Length of admission**					0.495
1–3 days	2 (22.2%)	3 (75.0%)	3 (33.3%)		
4–7 days	5 (55.6%)	1 (25.0%)	3 (33.3%)		
Over 7 days	2 (22.2%)	0 (0.0%)	3 (33.3%)		
**Outcome**					0.604
Favourable	6 (66.7%)	3 (75.0%)	7 (77.8%)		
Death	1 (11.1%)	1 (25.0%)	1 (11.1%)		
Discharged against medical advice	2 (22.2%)	0 (0.0%)	1 (11.1%)		

**Table 4:** ECG arrhythmias and conduction disorders in patients with acute CO poisoning.

		Severity of Poisoning			*p*-Value
		Mild Poisoning (*n* = 29)	Moderate Poisoning (*n* = 13)	Severe Poisoning (*n* = 23)	
**ECG**	Arrhythmias	5 (17.2%)	1 (7.7%)	2 (8.7%)	0.552
	Conduction disorders	5 (17.2%)	2 (15.4%)	4 (17.4%)	0.986

## Data Availability

Please contact the corresponding author for any supplement data.
